# Genome Sequence of *Aspergillus flavus* NRRL 3357, a Strain That Causes Aflatoxin Contamination of Food and Feed

**DOI:** 10.1128/genomeA.00168-15

**Published:** 2015-04-16

**Authors:** William C. Nierman, Jiujiang Yu, Natalie D. Fedorova-Abrams, Liliana Losada, Thomas E. Cleveland, Deepak Bhatnagar, Joan W. Bennett, Ralph Dean, Gary A. Payne

**Affiliations:** a J. Craig Venter Institute, Rockville, Maryland, USA; b Southern Regional Research Center, USDA/ARS, New Orleans, Louisiana, USA; c Rutgers University, School of Environmental and Biological Sciences, New Brunswick, New Jersey, USA; d Department of Plant Pathology, North Carolina State University, Raleigh, North Carolina, USA

## Abstract

Aflatoxin contamination of food and livestock feed results in significant annual crop losses internationally. *Aspergillus flavus* is the major fungus responsible for this loss. Additionally, *A. flavus* is the second leading cause of aspergillosis in immunocompromised human patients. Here, we report the genome sequence of strain NRRL 3357.

## GENOME ANNOUNCEMENT

Filamentous fungi destroy about 10% of the world’s crop harvest by contaminating food and livestock feed with mycotoxins. *Aspergillus flavus* in particular produces aflatoxins, which are the most potent naturally produced liver carcinogens. In addition to damaged human health in developing countries, the economic losses due to aflatoxin contamination in U.S. corn alone amounts to $280 million annually. Including cotton, peanut, and tree nuts, the economic losses are estimated to be more than 1 billion dollars in the United States. *Aspergillus flavus* spores are ubiquitously present in the air, soil, plant debris, and harvested grains.

*Aspergillus flavus* produces aflatoxins B1 and B2 and causes aflatoxin contamination of preharvest crops and postharvest grains during storage (1). The establishment of the aflatoxin biosynthesis pathway and the identification of biosynthesis gene clusters in *A. flavus* has been accomplished (2). The genome sequence of NRRL 3357 has resulted in genome-based efforts to understand the regulation of aflatoxin biosynthesis in order to discover new control strategies for the management of aflatoxin contamination (3).

The genome sequence of *A. flavus* NRRL 3357 was determined using the whole-genome shotgun method as described previously (4). Random shotgun libraries of 2- to 3-kb and 8- to 12-kb insert sizes were constructed from genomic DNA; DNA template was prepared for high-throughput sequencing using the ABI 3730XL instrument. Sequence reads were assembled using Celera Assembler.

Paired-end Sanger sequence reads provided 5× genome coverage, which was assembled into 958 contigs comprising 331 scaffolds ranging in size from 4.46 Mbp to 211 bp and containing a total of 958 contigs. The genome size is just under 37 Mbp, the scaffold *N*_50_ was 2.39 Mb, while the contig *N*_50_ was 103,582 bp. The 16 largest scaffolds, all greater than 350 kb, contained 98.8% of the genome, likely corresponding to the eight chromosomal arms. The average GC content was 44%. Genes were annotated using the JCVI eukaryotic annotation pipeline as described previously (5). The number of predicted protein-coding genes is 13,485. We determined the number of secondary metabolite biosynthetic gene clusters in the genome using the informatics tool SMURF (6). Fifty-six putative clusters were found for this strain, suggesting that the fungus is capable of producing a great many more compounds than just the aflatoxins.

### Nucleotide sequence accession number.

The annotated genome sequence of the *A. flavus* NRRL 3357 has been deposited at NCBI under whole-genome sequencing (WGS) accession number EQ963472.
